# Reliability of dopamine transporter PET measurements with [^18^F]FE-PE2I in patients with Parkinson’s disease

**DOI:** 10.1186/s13550-020-00676-4

**Published:** 2020-08-14

**Authors:** Vera S. Kerstens, Patrik Fazio, Mathias Sundgren, Granville J. Matheson, Erika Franzén, Christer Halldin, Simon Cervenka, Per Svenningsson, Andrea Varrone

**Affiliations:** 1 Centre for Psychiatry Research, Department of Clinical Neuroscience, Karolinska Institutet, and Stockholm Health Care Services, Region Stockholm, Karolinska University Hospital, Stockholm, Sweden; 2 Department of Clinical Neuroscience, Division of Neuro, Karolinska Institutet, Stockholm, Sweden; 3 Neurology Department, Karolinska University Hospital, Stockholm, Sweden; 4 Department of Neurobiology, Care Sciences and Society, Division of Physiotherapy, Karolinska Institutet, Stockholm, Sweden; 5 Function Area Occupational Therapy & Physiotherapy, Allied Health Professionals Function, Karolinska University Hospital, Stockholm, Sweden

**Keywords:** Reliability, Test-retest, [^18^F]FE-PE2I, Dopamine transporter, Parkinson’s disease

## Abstract

**Background:**

Reliable quantification of dopamine transporter (DAT), a biomarker for Parkinson’s disease (PD), is essential for diagnostic purposes as well as for evaluation of potential disease-modifying treatment. Due to degeneration of dopaminergic neurons and thus lower expected radioligand binding to DAT, higher measurement variability in PD patients might be expected than earlier reproducibility results in healthy controls. Therefore, we aimed to examine the test-retest properties of [^18^F]FE-PE2I-PET in PD patients.

**Methods:**

Nine patients with PD (Hoehn and Yahr stage < 3) were included (men/women 6/3; mean age 65.2 ± 6.8 years). Each patient underwent two [^18^F]FE-PE2I-PET measurements within 7–28 days. The outcome measure was non-displaceable binding potential generated using wavelet-aided parametric imaging with cerebellum as reference region. We assessed test-retest performance using estimates of reliability and repeatability. Regions for primary analysis were caudate, putamen, ventral striatum, and substantia nigra. Exploratory analysis was performed for functional subdivisions of the striatum. We also compared the more vs. less affected side.

**Results:**

[^18^F]FE-PE2I showed absolute variability estimates of 5.3–7.6% in striatal regions and 11% in substantia nigra and ICCs of 0.74–0.97 (median 0.91). The absolute variability for functional striatal subdivisions was 6.0–9.6% and ICCs of 0.76–0.91 (median 0.91). The less affected substantia nigra exhibited greater consistency than the more affected side. According to power calculations based on the current sample size, DAT changes of 5–11% in the striatum and 28% in the substantia nigra can be detected with a power of 0.8 (*p* < 0.0125).

**Conclusion:**

DAT-PET measurements with [^18^F]FE-PE2I in PD patients showed good repeatability and reliability. The slightly lower reliability in the substantia nigra in patients may be explained by lower DAT density and smaller anatomical size. Power calculations suggest that [^18^F]FE-PE2I PET is a suitable marker for longitudinal DAT decline in PD.

**Trial registration:**

EudraCT 2017-003327-29

## Background

Parkinson’s disease (PD) is a movement disorder characterized by progressive degeneration of the dopaminergic system, affecting both the cell bodies in the substantia nigra and its projections to the striatum. Within the dopaminergic system, the dopamine transporter regulates the synaptic dopamine levels by dopamine reuptake, and its density reflects presynaptic functioning.

[^18^F]FE-PE2I, developed in 2009 [1, 2], has, through several validity studies, proven to be a valuable positron emission tomography (PET) radioligand for dopamine transporter (DAT) imaging [1–8]. A clinical PET study with [^18^F]FE-PE2I in twenty patients with early-stage PD showed an in vivo striato-nigral gradient of DAT loss [9], in agreement with post-mortem studies in patients and animal model studies [10, 11]. After initial clinical validation [6], additional studies showed that simplified quantification of [^18^F]FE-PE2I-PET can be achieved with a shortened imaging protocol, making clinical implementation realistic [7, 12]. The test-retest reliability of [^18^F]FE-PE2I has been previously studied in twelve young, healthy men [13], showing low variability and good reliability. However, in order to evaluate the suitability of [^18^F]FE-PE2I as a disease progression marker, it is critical to assess reliability also in patient samples. Due to the degeneration of striatal projections, patients with PD have lower DAT availability, which could lead to lower measurement reliability compared with healthy controls.

In the majority of PET studies of the striatal dopaminergic system, the regions of interest are the striatum, divided into caudate, putamen, and ventral striatum (nucleus accumbens), and the substantia nigra (midbrain). This anatomical subdivision of the striatum, although useful, might not represent the functional organization of the striatum. Instead, subdivision based on the connectivity between the basal ganglia and the neocortex can be used, where the striatum is divided into a limbic, associative, and sensorimotor striatum (respectively ventral striatum, caudate and ventrolateral putamen, and dorsolateral putamen) [14]. The functional regions have shown to be useful for molecular imaging studies examining correlations with behavioral and clinical outcome measures [15–17].

The assessment of the test-retest reliability of DAT-PET in patients with PD is relevant for several reasons. First, the knowledge on the natural variability is essential for interpretation of longitudinal follow-up study results; second, the measured variability can be used to estimate the minimum effect size on DAT needed for disease-modifying treatment trials; and third, for the purpose of power calculations for future clinical studies investigating longitudinal treatment efficacy.

The primary objective of this study was, therefore, to assess the test-retest reliability of [^18^F]FE-PE2I measurements in the main striatal areas and substantia nigra in patients with PD. The hypothesis was that the reliability in the striatum would be similar to that observed in healthy subjects, and the reliability in the substantia nigra lower than in the striatum considering the lower DAT density in the substantia nigra. The secondary objective was to evaluate the test-retest reliability of three connectivity-based functional subdivisions of the striatum in view of future PET analyses.

## Materials and methods

### Study population

Eleven patients with PD, Hoehn and Yahr (H&Y) stage < 3, were recruited via advertisement on the Swedish Parkinson Foundation website and via two specialist outpatient clinics in Stockholm (Academic Specialist Centre, Karolinska University Hospital). None of the subjects had clinically relevant somatic comorbidities, cognitive decline, history of psychiatric disease, illicit drug abuse, or alcoholism, as assessed by a structured interview. Physical examination, electrocardiography, and routine blood tests were normal. One patient had to be excluded from the PET analysis because the cerebellum was partly out of the PET axial field of view. Demographic details are shown in Table 1.

**Table 1:** Demographic and clinical characteristics of the patients

Subject	Age (years)/sex	Symptom duration (years)	MDS-UPDRS-III		H&Y	LEDD (mg)^a^	Days between PET 1–2	Avg. steps/day^b^		Avg. magnitude counts/day^b^	
			PET1	PET2				PET1	PET2	PET1	PET2
1	67/F	8	16	16	1	425	7	5133	6925	296,027	390,733
2	68/M	7	10	9	1	500	7	11,904	7254	584,009	369,687
3	56/F	14	16	21	1	680	7	6496	4429	345,169	335,207
4	71/M	5	18	28	1	560	7	4361	4770	261,553	275,948
5	69/M	8	30	30	2	675	20	5428	6854	340,436	403,469
6	54/M	3	10	7	2	650	28	7232	6930	422,193	368,935
7	74/M	8	25	20	2	400	7	3976	6152	267,269	350,235
8	47/M	2.5	3	6	1	320	17	7084	7166	501,065	524,408
9	67/F	5	40	36	2.5	300	7	3091	5971	248,016	323,042
10	61/M	2.5	16	16	2	150	14	12,020	11,801	676,188	857,889
Mean ± SD	63.4 ± 8.6	6.3 ± 3.5	18.2 ± 10.8	18.7 ± 10.2		465 ± 180	–	–		–	
Median					1.5		7	5962	6889	342,803	369,311

*MDS-UPDRS-III* Movement Disorder Society Unified Parkinson’s Disease Rating Scale, part 3, motor function (range 0–72). Assessed before PET1 and PET2, respectively; *H&Y* Hoehn and Yahr stage (range 1–5); *LEDD* levodopa equivalent daily dose

^a^No changes in LEDD have occurred between PET assessments

^b^Assessed with the activity monitor (Actigraph GT3X+)

### Data collection

#### Activity monitor and disease severity assessment

An activity monitor (Actigraph GT3X+) was worn on the hip for 5–7 days before each PET measurement. Average amount of steps and magnitude of movement per day were measured as a supportive measure of clinical motor stability [18, 19]. Only days with minimal 540 min wear time were included in the calculation [20]. As measure of disease severity, the Movement Disorder Society Unified Parkinson’s Disease Rating Scale, part 3, motor function (MDS-UPDRS-III) was done, including H&Y staging. All MDS-UPDRS-III assessments were performed on the same time of day by the same physician (VSK) in practically defined “OFF” (see below). Symptom duration was defined as the time from reported onset of first motor symptoms.

#### MRI acquisition

Using a 3 Tesla MRI system (General Electric, Discovery MR750), T2-weighted images were acquired to exclude clinically significant pathology, and 3D T1-weighted images were acquired for co-registration with PET and delineation of the regions of interest (ROI). This last sequence has 176 slices of 1 mm thickness, field of view 256 × 256 mm, resolution 1 × 1 × 1 mm, inversion time 450 ms, echo time 3.18 ms, and repetition time 8.16 ms.

#### PET acquisition

[^18^F]FE-PE2I was prepared as previously described [21]. Two 93-min [^18^F]FE-PE2I PET measurements were acquired in each subject within an interval of 7–28 days (see PET injection characteristics, Supplementary Table S1). PET measurements were done on the same time of day, around 1:30 pm. Patients were asked to be practically defined “OFF,” meaning a withdrawal of levodopa-medication for at least 12 h and other dopaminergic medication for at least 24 h. Also, abstinence of caffeine 3 h before PET, nicotine on day of PET, alcohol 48 h before PET, and cardiovascular training 96 h before PET were requested. An individually made plaster helmet was used for head fixation in the PET camera. PET measurements were acquired with a high-resolution research tomograph (HRRT, Siemens Molecular Imaging) after an intravenous bolus injection of [^18^F]FE-PE2I. Details can be found in Supplementary Table S1. A 6-min transmission scan with a Caesium-137 source was obtained for attenuation correction. Due to technical reasons, the transmission scan for one patient could not be acquired on the day of first PET measurement, so the transmission scan acquired before the second PET measurement was used for attenuation correction of the first PET measurement.

List mode PET data were reconstructed into 37 frames (8 × 10, 5 × 20, 4 × 30, 4 × 60, 4 × 180, 12 × 360 s) using 3D OP OSEM with 10 iterations and 16 subsets, including modeling of the PSF [22]. Frame-to-frame realignment was performed as previously described [23], with the only difference that the first 2 min instead of the first minute were used as reference frame for PET realignment.

#### PET motion correction

Head motion was evaluated by patient observation during data acquisition as well as during image analysis by reviewing the realignment plots and brain time activity curves (TACs). Translation of more than 3 mm on the realignment plots led to additional motion correction using an in-house developed automatic procedure. Description of the method is given in Supplementary Text 1.

### Image analysis

Using SPM12, the T1-weighted 3D MRI sequence was first realigned to the AC-PC plane (anterior commissure-posterior commissure), after which the PET was realigned and co-registered to the realigned MRI. The following regions of interest were then delineated automatically on the T1-weighted images with FreeSurfer version 6.0.0 (http://surfer.nmr.mgh.harvard.edu/): whole striatum (STR), caudate (CAU), putamen (PUT), ventral striatum (VS), and cerebellum. For substantia nigra (SN), the functional molecular template, as created in the research group [9], was used. As exploratory outcome, three functionally subdivided striatal areas [14] were added to the analysis (http://fsl.fmrib.ox.ac.uk/fsl/fslwiki/Atlases/striatumconn). For all regions, regional non-specific binding potentials (*BP*_ND_) of [^18^F]FE-PE2I were generated using wavelet-aided parametric imaging (WAPI) [24] with t* = 27 min and the cerebellum as reference region.

As described above, for one subject, only one transmission scan could be acquired, which had to be used for attenuation correction of both PETs. This technical issue introduced a bias in *BP*_ND_ due to misaligned attenuation correction in the reference region. See Supplementary Text 2 for analysis and explanation. It was, therefore, decided to exclude the subject for the main analysis and report the results including the outlier as supplementary material. We believe that the test-retest metrics calculated without the outlier are more representative of the study sample.

### Statistical analysis

For statistical analysis, R version 3.4.3 was used with the package *relfeas* (https://github.com/mathesong/relfeas). [^18^F]FE-PE2I measurement reproducibility was determined with calculation of repeatability (absolute intrasubject variability, AbsVar; and the minimum detectable difference, MDD) and reliability (intraclass correlation coefficient, ICC), as per recommendation of Weir, Baumgartner, and Matheson [25–27]. Absolute variability was calculated as: (test–retest)/(mean test and retest) × 100. For ICC, the two-way random effects, absolute agreement, single rater/measurement was used, corresponding to:

$$ \frac{{\mathrm{MS}}_{\mathrm{S}}\hbox{-} {\mathrm{MS}}_{\mathrm{E}}}{{\mathrm{MS}}_{\mathrm{S}}+\left(k\hbox{-} 1\right){\mathrm{MS}}_{\mathrm{E}}+k\left({\mathrm{MS}}_{\mathrm{T}}\hbox{-} {\mathrm{MS}}_{\mathrm{E}}\right)/n} $$

with MS_S_, subjects mean square; MS_E_, error mean square; *k*, number of trials; MS_T_, trials mean square; and *n*, number of subjects.

The ICC represents the proportion of the variability not attributable to measurement error. As such, an ICC of 1 indicates perfect measurement reliability with all observed variability being due to true (biological) differences and none to measurement variability (error), while an ICC of 0.5 indicates that the variability is comprised of true differences and measurement error in equal measure. Different interpretations of the ICC exist; as proposed by Portney and Watkins [28] and suggested by Matheson [27], we regard an ICC < 0.5 as low, 0.5–0.75 moderate, 0.75–0.9 good, and > 0.9 excellent. Measurement reliability with an ICC > 0.9 is recommended as a lowest acceptable standard for measurements from which diagnostic decisions are made, ICC > 0.7 for research purposes, with 0.95 and 0.8 considered as adequate, respectively [29].

The agreements between measurements in each region were plotted with the Bland-Altman plots. Power plots were generated with the jamovi software (https://www.jamovi.org/). The results of study variables are expressed as mean ± standard deviation unless otherwise stated.

## Results

All subjects completed the study according to the protocol, with exception of the subject with only one transmission acquisition. The MDS-UPDRS-III and Actigraph outcomes support the subject’s clinical stability during the study period (Table 1), with the exception of two patients who had a week of influenza and a week of holiday respectively explaining the lower Actigraph outcome. One subject showed a large difference in test/retest MDS-UPDRS-III (18 vs. 28), based on 1 point increases spread over the different domains, with the left side being mildly symptomatic in the second assessment versus not symptomatic in the first assessment. The difference could not be explained by patient self-report and is probably due to either natural symptom fluctuations and/or intra-observer variability.

**Fig. 1:**
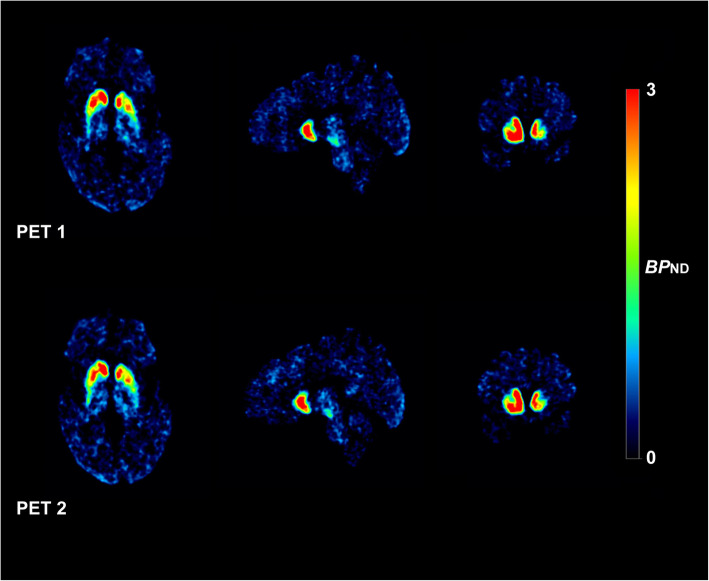
Representative test and retest parametric *BP*_ND_ images of [^18^F]FE-PE2I

**Fig. 2:**
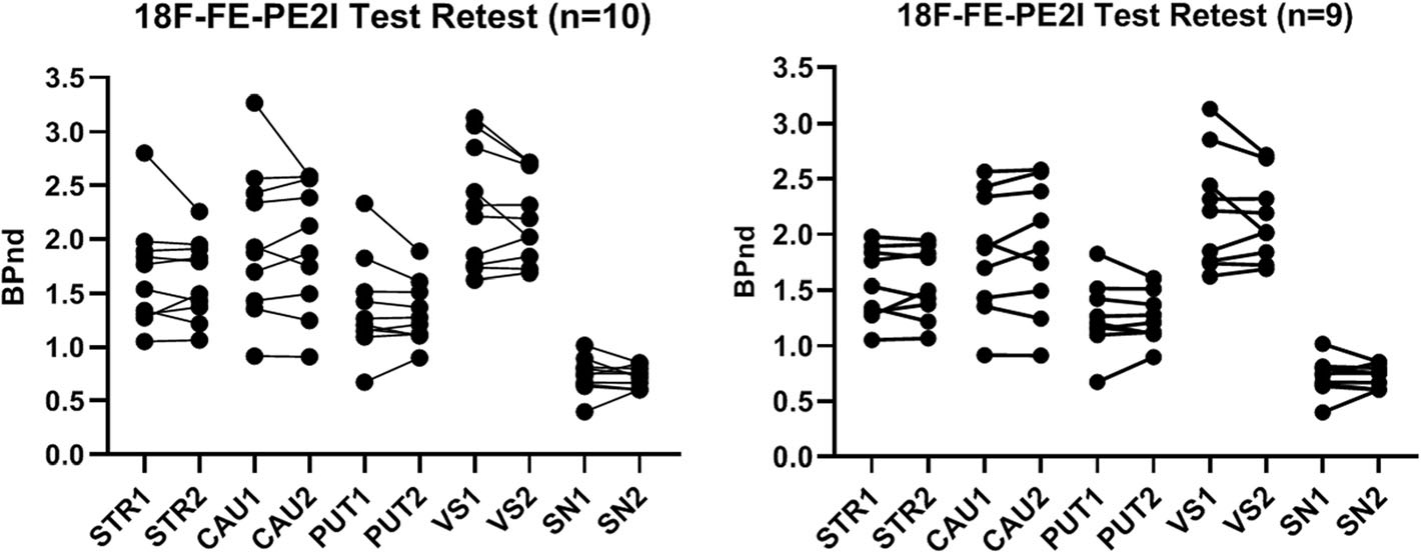
Individual *BP*_ND_ values between scan 1 and scan 2. Striatal regions and substantia nigra, with and without the exclusion of the outlier, are displayed

**Fig. 3:**
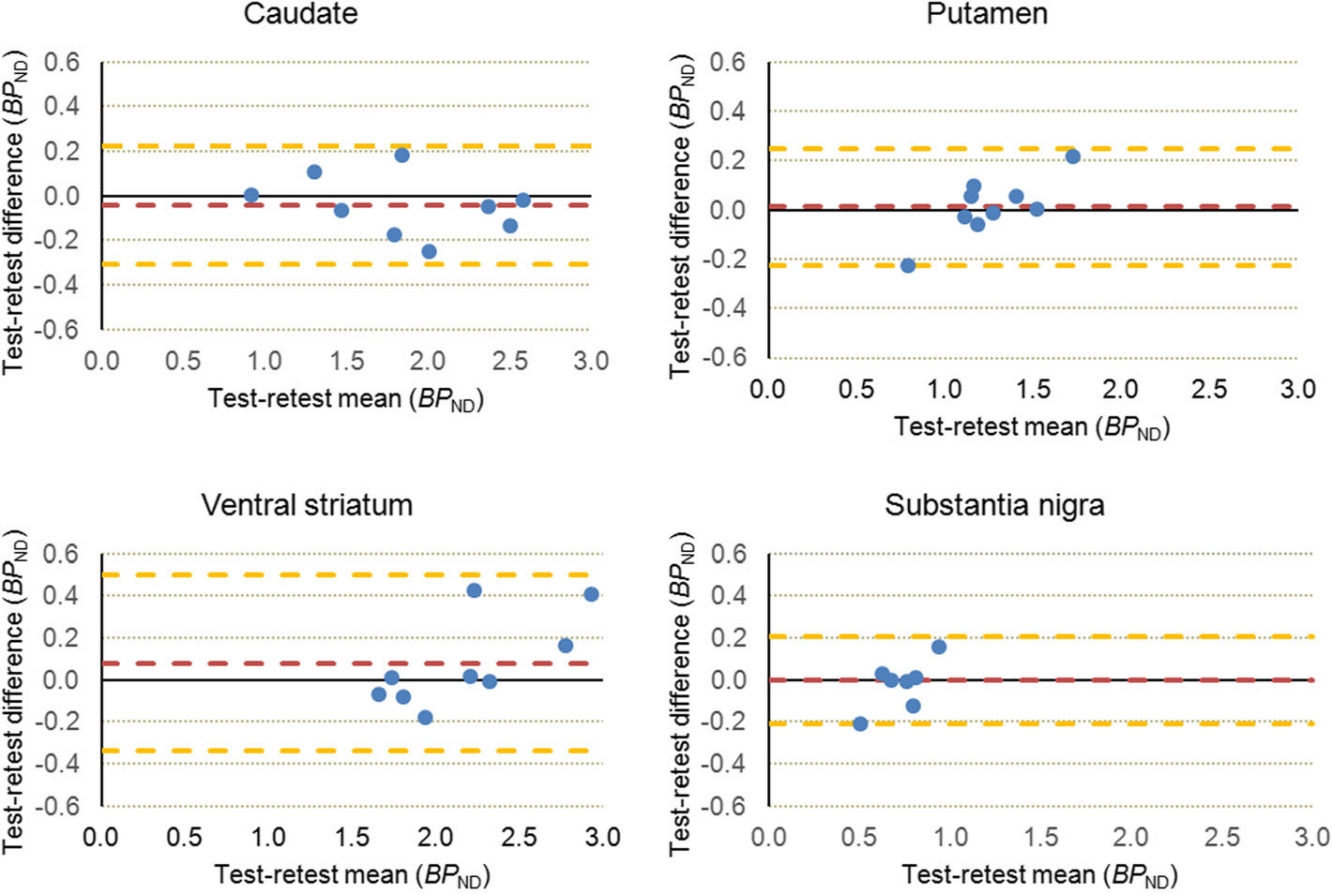
Bland-Altman plots of main regions of interest. The yellow lines correspond to the upper and lower 2SD line; red line: bias

Representative test-retest *BP*_ND_ images of [^18^F]FE-PE2I are shown in Fig. 1. Table 2 shows the individual test-retest *BP*_ND_ values for the four main regions of interest, with the highest *BP*_ND_ in the CAU and VS and lowest in PUT and SN (Fig. 2). The Bland-Altman plots (Fig. 3) showed good agreement between the test-retest measurements. Supplementary Figure S1 shows the Bland-Altman plots including the outlier described earlier. Test-retest calculations are reported in Table 3. The striatal regions displayed low variability (AbsVar 5.3–7.5%) and high ICC (0.89–0.97). SN showed relatively higher absolute variability (11%), with a moderate ICC (0.74).

**Table 2:** Individual binding potential (*BP*_ND_) values of [^18^F]FE-PE2I in striatal regions and substantia nigra

	Striatum		Caudate		Putamen		Ventral striatum		Substantia nigra	
Subject	PET 1	PET 2	PET 1	PET 2	PET 1	PET 2	PET 1	PET 2	PET 1	PET 2
1	1.763	1.827	2.428	2.562	1.264	1.275	1.736	1.722	0.73	0.849
2	1.051	1.065	0.914	0.91	1.095	1.121	1.623	1.687	0.635	0.602
3	1.892	1.91	2.339	2.388	1.516	1.513	2.317	2.319	0.813	0.798
4	1.835	1.789	1.697	1.872	1.824	1.607	3.131	2.717	1.016	0.854
5	1.306	1.372	1.43	1.496	1.15	1.21	1.848	2.021	0.668	0.666
6	1.273	1.497	1.876	2.125	0.672	0.898	1.758	1.838	0.397	0.602
7	1.339	1.217	1.356	1.245	1.203	1.104	2.441	2.011	0.752	0.754
8	2.801	2.256	3.268	2.588	2.332	1.888	3.054	2.714	0.894	0.716
9	1.537	1.426	1.928	1.744	1.166	1.112	2.211	2.191	0.653	0.603
10	1.978	1.948	2.564	2.582	1.423	1.37	2.855	2.686	0.796	0.75

*PET 1* test, *PET 2* retest

**Table 3:** Test-retest metrics of [^18^F]FE-PE2I PET measurements (*n* = 9)

Region	PET 1 (*BP*_ND_), COV (%)	PET 2 (*BP*_ND_), COV (%)	AbsVar (%)	ICC	MDD	Powered detectable % change
Striatum	1.55 ± 0.33, 21.1	1.56 ± 0.32, 20.5	5.3	0.95 (0.82–0.989)	0.195	− 8.3
Caudate	1.84 ± 0.55, 29.9	1.88 ± 0.59, 31.4	6.0	0.97 (0.89–0.99)	0.264	− 8.9
Putamen	1.26 ± 0.32, 25.3	1.25 ± 0.22, 17.8	7.5	0.91 (0.68–0.98)	0.225	− 11.9
Ventral striatum	2.21 ± 0.53, 23.9	2.13 ± 0.38, 17.9	6.5	0.89 (0.61–0.97)	0.426	− 12.1
Substantia nigra	0.72 ± 0.17, 23.2	0.72 ± 0.1, 14.5	10.6	0.74 (0.24–0.93)	0.194	− 17.9

*COV* coefficient of variability (SD/mean × 100), *AbsVar* absolute variability, *ICC* intraclass correlation coefficient, *MDD* minimum detectable difference covering 95% of the distribution of test-retest differences, *Powered detectable % change* based on measured effect size and power 0.8

Bland-Altman plots of the exploratory regions of interest are presented in Supplementary Figure S2, and the repeatability and reliability results in Table S3. Low absolute variability and good ICC (> 0.75) were observed in the functional striatal subdivisions.

For transparency, test-retest metrics calculated including the outlier are reported in Supplementary Table S2.

Additionally, the reliability was assessed for the outcomes of the less vs. more affected hemisphere (Supplementary Table S3). This analysis showed similar test-retest consistency for both the more and less affected hemispheres. The less affected SN exhibited numerically better reliability and repeatability, although not significant.

## Discussion

This study was designed to assess the test-retest reproducibility of [^18^F]FE-PE2I PET-measurements of DAT in Parkinson patients (H&Y stage < 3). The results showed good repeatability and reliability of the measurements, providing support that [^18^F]FE-PE2I can be used as DAT biomarker in PD.

The [^18^F]FE-PE2I test-retest study in twelve young, healthy controls [13] observed comparable repeatability (CAU 4.8%, PUT 5.6%, SN 9.7%) and reliability (CAU 0.83, PUT 0.88, SN 0.71). This shows that the lower DAT availability as consequence of PD, at least in H&Y stages < 3, does not substantially influence the consistency of its measurements*.* Relatively higher ICC in patient cohorts compared to healthy control cohorts is to be expected because of inherently higher inter-individual differences in patient cohorts than in healthy control cohorts.

### Test-retest reproducibility of other DAT-PET and DAT-SPECT radioligands

Hirvonen et al. [30] showed in their same-day test-retest study in five healthy subjects an absolute variability and reliability of ^11^C-PE2I measurements in manually delineated ROIs for ventral striatum and midbrain of 7.2 ± 4.4% and 6.5 ± 5.2%, and ICC of 0.81 and 0.83, respectively. The higher ICC in midbrain compared to Suzuki et al. is probably due to higher absolute *BP*_ND_ values in the midbrain observed for ^11^C-PE2I and measured with an HRRT. Nurmi et al. [31] found the intraclass correlations of ^18^F-CFT uptake values in eight healthy controls to be 0.91, 0.94, and 0.86 for caudate, anterior putamen, and posterior putamen, respectively (manual ROIs). In the seven de novo PD patients, the intraclass correlation was 0.97, 0.95, and 0.96, respectively, which is comparable to our results. Scans were performed 2.5–3 months apart, with the second scan in PD patients being after a 3-month levodopa treatment, showing that even after initiated levodopa treatment, the DAT-measurements have high reproducibility in PD patients in this time range.

Test-retest results of clinical DAT-SPECT radioligands ^123^I-beta-CIT and ^123^I-FP-CIT showed measurement variability in PD patients of 7.4–16.8 % in STR and 12.2% in striatal subdivisions [32–34], with corresponding ICCs of 0.59–1.00 [33, 34]. Results vary because of different ROI definition methods and outcome measures. The striatal test-retest variability of ^123^I-PE2I in seven healthy subjects [35] was 5.2 ± 4.5 (STR), 9.4 ± 7.0 (CAU), and 10.3 ± 5.1% (PUT), with corresponding ICC of 0.92, 0.83, and 0.84. Our test-retest results are thus comparable to the clinically implemented DAT-SPECT radioligands. Given the advantages of PET compared to SPECT, the results confirm that ^18^F-FE-PE2 is a strong candidate for clinical applications as well.

### DAT measurement in smaller regions

The higher resolution of PET compared with SPECT permits a better assessment of low-binding regions, such as the SN. Test-retest repeatability and reliability in this region were inferior to test-retest metrics in the striatum. The smaller size of the SN and the smaller numerical value of *BP*_ND_ in this region are likely reasons for its greater variability. Despite this relative limitation, a previous study on DAT availability in the nigrostriatal system of early PD patients as quantified with [^18^F]FE-PE2I [9] showed changes of DAT availability in the SN compared to healthy controls that were still larger (30%) than the reliably detectable difference (~ 18%, based on effect size of *n* = 9), confirming that the assessment of the nigrostriatal system with [^18^F]FE-PE2I PET provides a comprehensive assessment of the PD pathophysiology in vivo.

The low variability and high reliability observed in the connectivity-based functional subdivisions of the striatum furthermore indicate that [^18^F]FE-PE2I PET is a reliable research tool. This is relevant for studies using the functional rather than anatomical striatal subdivisions in assessing correlations of DAT availability to specific clinical variables of cognitive function or behavior.

### Power calculation

**Fig. 4:**
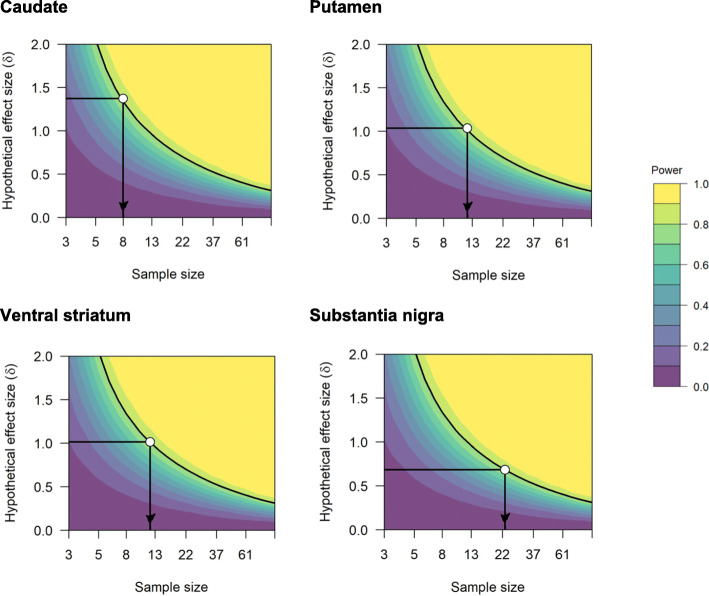
Relationship between sample size and effect size for caudate, putamen, ventral striatum, and substantia nigra. The arrow indicates the sample size needed to detect a statistically significant difference with 0.8 power, based on the effect size corresponding to a 10% change

Using the variability in test-retest differences within each region, we estimated the size of within-individual changes which could be detected with a power of 80%, using a significance threshold of 0.0125 (making use of Bonferroni correction for multiple comparisons for the four primary regions of interest, 0.05/4). These estimates suggest that a study with a sample size of 9 patients is sufficiently sensitive to detect within-individual differences of greater than between 5 and 11% for different regions within the striatum and greater than 28% for the SN (Supplementary Table S2). The relationship between sample size and effect size was examined as a function of statistical power and is presented in Fig. 4. Yearly DAT decline in PD patients has been estimated to be between 5 and 13% in striatal regions [31, 36, 37], meaning that [^18^F]FE-PE2I PET is well suited for measuring biological DAT differences in striatal regions in a longitudinal follow-up study with a typical sample size for PET studies.

## Conclusion

[^18^F]FE-PE2I measurements of DAT have good reliability in Parkinson patients (H&Y 1–2.5) even in the small anatomical areas with lower DAT density, such as the substantia nigra. The test-retest metrics were equal-to-superior to other DAT radioligands. Thus, this study further supports the suitability of [^18^F]FE-PE2I as imaging marker for longitudinal follow-up studies in PD.

## Supplementary information

**Additional file 1:** Supplementary material.

## Data Availability

The dataset of the current study is available from the corresponding author on reasonable request.

## Abbreviations

[18F]FE-PE2I: 18F-(E)-N-(3-Iodoprop-2-Enyl)-2b-Carbofluoroethoxy-3b-(49-Methyl-Phenyl) Nortropane; 3D OP OSEM Three dimensional ordered Poisson ordered subset expectation-maximization
AbsVar: Absolute variability
AC-PC: Anterior commissure-posterior commissure
BPnd: Non-displaceable binding potential
CAU: Caudate
COV: Coefficient of variability
DAT: Dopamine transporter
H&Y: Hoehn and Yahr disease severity score
ICC: Intraclass correlation coefficient
LEDD: Levodopa equivalent daily dose
MDD: Minimum detectable difference
MDS-UPDRS-III: Movement Disorder Society Unified Parkinson’s Disease Rating Scale, part 3, motor function
MRI: Magnetic resonance imaging
PD: Parkinson’s disease
PET: Positron emission tomography
PSF: Point-spread function
PUT: Putamen
ROI: Region of interest
SN: Substantia nigra
SPECT: Single-photon emission computed tomography
SPM12: Statistical Parametric Mapping software, current version
STR: Striatum
TAC: Time activity curve
VS: Ventral striatum
WAPI: Wavelet-aided parametric imaging
