# Supplementary material for: Reliability of dopamine transporter PET measurements with [18F]FE-PE2I in patients with Parkinson’s disease
Source: EJNMMI Res. 2020 Aug 14;10:95. doi: 10.1186/s13550-020-00676-4 (PMC7427674; doi:10.1186/s13550-020-00676-4)
Supplement: Supplementary file 1 — Additional file 1: Supplementary material. [file 13550_2020_676_MOESM1_ESM.pdf]

SUPPLEMENTARY TEXT

Motion correction procedure

First, non-attenuation corrected emission frames were co-registered to transmission data with SPM12. Then, the transformation parameters were applied to the attenuation map, creating attenuation data aligned with the emission data. Then a new reconstruction was performed by using the co-registered attenuation map. Lastly, motion-corrected frames were realigned to initial position using the inverse of the co-registered parameters estimated in the first step.
If this motion correction did not improve the time activity curves as assessed visually, either the non-motion corrected PET was used in case that time activity curve was deemed as good enough, or in case of suspected in-frame motion that could not be corrected by the automated motion correction, additional analysis of the involved frame was done by extracting the TAC after exclusion of the suspected frame. In six cases, motion correction of the PET had to be applied; three directly after PET acquisition based on observed in-camera motion (P7[2],P10[1], P10[2]), three after inspection of the TACs after realignment (P1[2], P8[1], P9[1]). The TAC of one of the former and two of the latter did not improve after automated frame-by-frame motion correction, suggesting in-frame motion. Additional inspection led to two scans finally being analyzed with a removed frame (frame 28 in P8[1], frame 31 in P9[1]), and one analyzed with all frames kept (P10[1]).

Outlier analysis

To understand whether the bias in the BP_{ND}-estimation in the outlier was related to misaligned attenuation correction in the cerebellum, a post-hoc analysis was done. The hypothesis was that a slight misalignment of the attenuation map might have led to underestimation of radioactivity concentration in the reference region (cerebellum) causing overestimation of BP_{ND} in the regions of interest (see supplementary figure S3). For this, SUV and AUC of the striatum and cerebellum were calculated for all subjects. The results showed that in P8 the AUC of the striatum in PET 1 was 5.88% higher than the AUC of the striatum in PET 2, whereas the AUC of the cerebellum was 6.98% *lower* in PET1 than PET2. This pattern was not observed in the remaining patients, supporting the hypothesis that the misaligned attenuation correction in the cerebellum contributed to the bias of BP_{ND} in P8.

SUPPLEMENTARY TABLES

SUPPLEMENTARY TABLE S1. Individual patients' weight and details of PET.

Subject	Weight Pre-PET1 (kg)	Injected radioactivity PET1 (MBq)	Molar activity (GBq/μmol)	Mass (μgr)	Weight Pre-PET2 (kg)	Injected radioactivity PET2 (MBq)	Molar activity (GBq/μmol)	Mass (μgr)
**1**	71.4	191	51	1.72	69.9	187	48	1.79
**2**	73.7	198	25	3.59	74.4	228	206	0.51
**3**	79.6	217	65	1.53	79.4	213	56	1.73
**4**	72.5	189	51	1.68	73.0	194	249	0.36
**5**	78.9	190	59	1.48	79.6	221	87	1.17
**6**	85.1	232	65	1.62	86.1	240	80	1,37
**7**	63.8	181	87	0.95	63.7	175	89	0.90
**8**	93.0	267	30	4.02	91.8	258	38	3.12
**9**	78.2	222	48	2.13	77.9	218	47	2.11
**10**	81.0	230	42	2.52	80.1	232	47	2.26
**Mean ± SD**	77.2 ± 8.0	211.7 ± 26.8	-	2.12 ± 0.98	77.6 ± 8.0	216.5 ± 25.3	-	1.53 ± 0.85
**Median**			51				68	

Median values are reported for data not normally distributed.

SUPPLEMENTARY TABLE S2. Test-retest metrics of ^{18}F-FE-PE2I PET measurements with inclusion of outlier subject (n = 10).

Region	PET 1 (BP_{ND})<br>COV (%)	PET 2 (BP_{ND})<br>COV (%)	AbsVar (%)	ICC	MDD	Powered detectable % change
**Striatum**	1.68 ± 0.50<br>29.9	1.63 ± 0.37<br>22.9	7.0	0.90 (0.67-0.97)	0.385	-13.9
**Caudate**	1.98 ± 0.69<br>34.7	1.95 ± 0.60<br>30.7	7.7	0.93 (0.74-0.98)	0.490	-15.2
**Putamen**	1.36 ± 0.45<br>33.2	1.31 ± 0.29<br>22.3	8.8	0.89 (0.64-0.97)	0.348	-15.2
**Ventral Striatum**	2.30 (0.56)<br>24.6	2.19 ± 0.40<br>18.5	7.0	0.89 (0.63-0.97)	0.456	-11.1
**Substantia nigra**	0.74 ± 0.17<br>22.7	0.72 ± 0.10<br>13.7	11.7	0.68 (0.15-0.91)	0.215	-17.9

COV: Coefficient of variability (SD/mean *100); **AbsVar:** Absolute variability; **ICC:** Intraclass correlation coefficient; **MDD**: Minimum detectable difference; **Powered detectable %change:** based on measured variability, power 0.8 and sample size = 10.

SUPPLEMENTARY TABLE S3. Test-retest results of the connectivity-based functional striatal subdivisions (n=9).

Region	PET 1 (BP_{ND})<br>COV (%)	PET 2 (BP_{ND})<br>COV (%)	AbsVar (%)	ICC	MDD	Powered detectable % change
**Limbic striatum**	2.04 ± 0.53<br>23.9	2.13 ± 0.38<br>17.9	10.3	0.91 (0.68-0.98)	0.472	-15.4
**Associative striatum**	1.64 ± 0.53<br>32.1	1.75 ± 0.47<br>27	12.8	0.91 (0.65-0.98)	0.412	-13.7
**Sensorimotor striatum**	0.79 ± 0.22<br>28.5	0.77 ± 0.17<br>22.2	16.4	0.76 (0.28-0.94)	0.269	-22.8

COV: Coefficient of variability (SD/mean *100); **AbsVar:** Absolute variability; **ICC:** Intraclass correlation coefficient; **MDD**: Minimum detectable difference; **Powered detectable % change:** based on measured variability, power 0.8, and sample size = 9.

SUPPLEMENTARY TABLE S4. Test-retest results less vs. more affected side (hemisphere) (n = 9).

Region	PET 1 (BP_{ND}) COV (%)	PET 2 (BP_{ND}) COV	AbsVar (%)	ICC	MDD	Powered detectable % change
**Less affected striatum**	1.72 ± 0.35<br>20.5	1.72 ± 0.36<br>21.0	6.3	0.94 (0.79-0.99)	0.235	-9.1
**More affected striatum**	1.39 ± 0.32<br>23.1	1.4 ± 0.3<br>21.3	5.3	0.96 (0.85-0.99)	0.168	-7.9
**Less affected caudate**	1.98 ± 0.57<br>28.6	2.03 ± 0.64<br>31.7	7.3	0.97 (0.87-0.99)	0.311	-9.7
**More affected caudate**	1.69 ± 0.55<br>32.5	1.73 ± 0.56<br>32.3	6.4	0.98 (0.91-0.995)	0.235	-8.7
**Less affected putamen**	1.45 ± 0.35<br>24.5	1.42 ± 0.26<br>18.5	7.7	0.92 (0.69-0.98)	0.253	-11.4
**More affected putamen**	1.07 ± 0.29<br>27.1	1.07 ± 0.19<br>18.2	8.0	0.91 (0.67-0.98)	0.207	-12.8
**Less affected ventral striatum**	2.25 ± 0.56<br>24.9	2.22 ± 0.44<br>19.8	8.9	0.89 (0.61-0.97)	0.468	-13.7
**More affected ventral striatum**	2.18 ± 0.54<br>24.7	2.06 ± 0.4<br>19.4	8.7	0.86 (0.52-0.97)	0.494	-13.6
**Less affected substantia nigra**	0.78 ± 0.15<br>19.2	0.77 ± 0.13<br>16.6	8.8	0.83 (0.45-0.96)	0.158	-13.5
**More affected substantia nigra**	0.66 ± 0.2<br>31.2	0.67 ± 0.12<br>18.7	16.0	0.64 (0.06-0.91)	0.277	-27.8

COV: Coefficient of variability (SD/mean *100); **AbsVar:** Absolute variability; **ICC:** Intraclass correlation coefficient; **MDD**: Minimum detectable difference; **Powered detectable %change:** based on measured effect size and power 0.8.

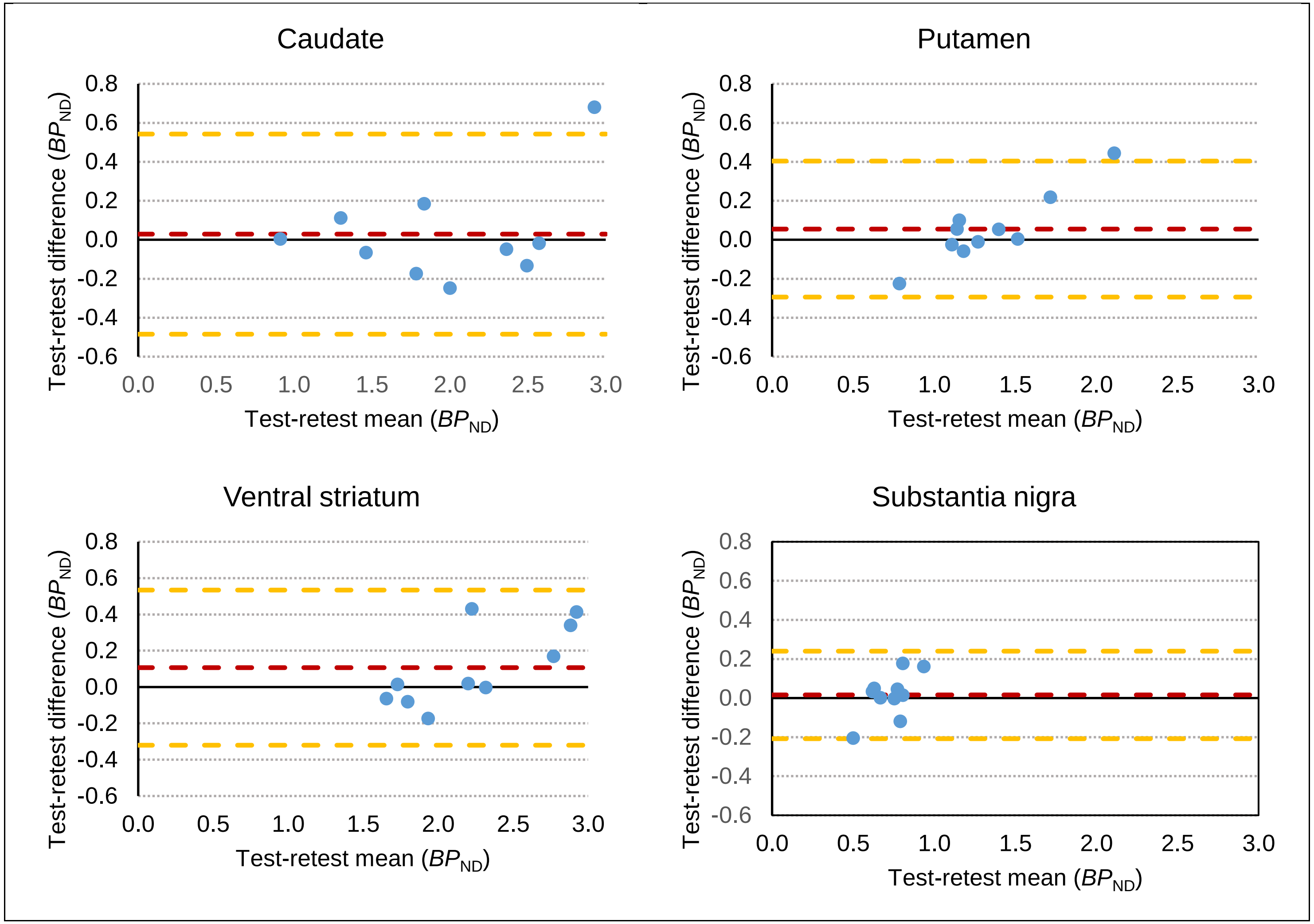

SUPPLEMENTARY FIGURE S1. Bland Altman plots including the outlier subject. Yellow lines: the upper and lower 2SD; red line: bias.

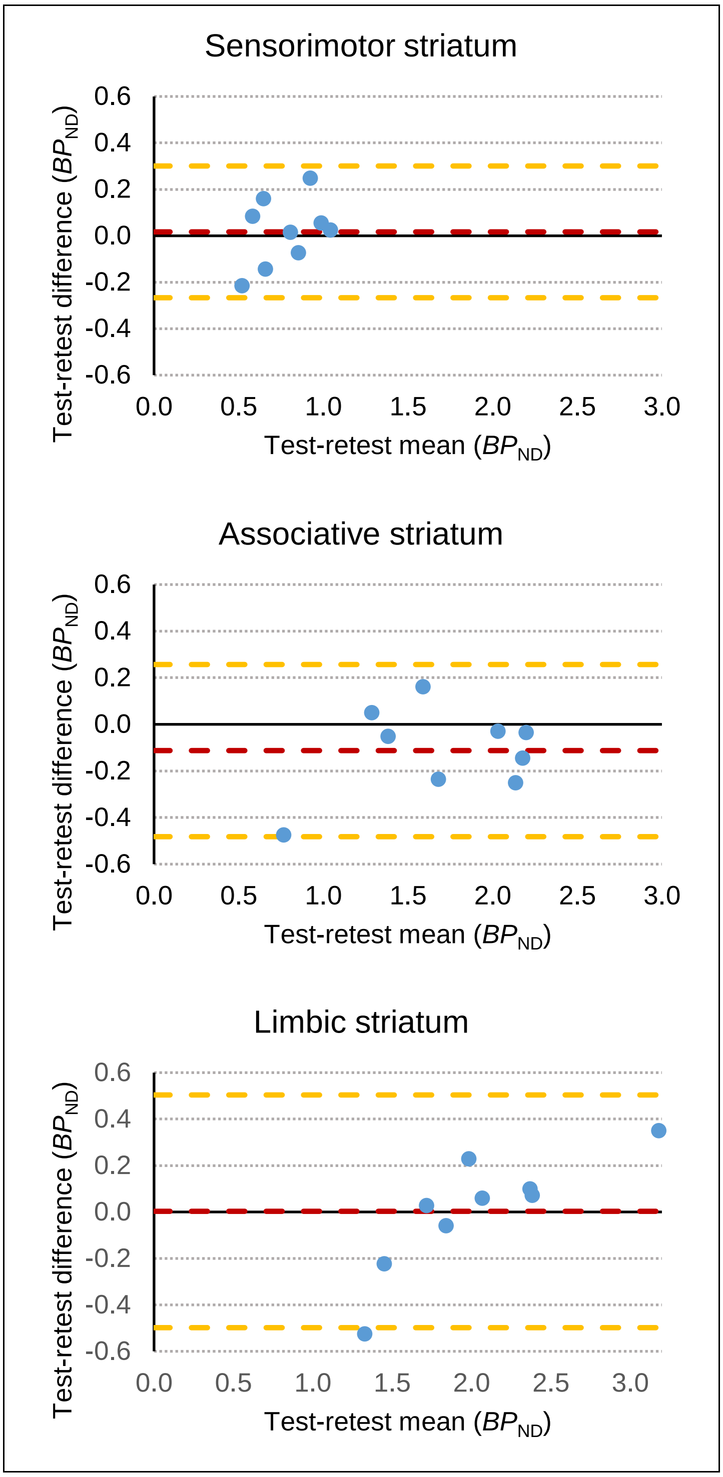

SUPPLEMENTARY FIGURE S2. Bland Altman plots of the functionally subdivided striatal regions. Yellow lines: the upper and lower 2SD line; red line: bias.

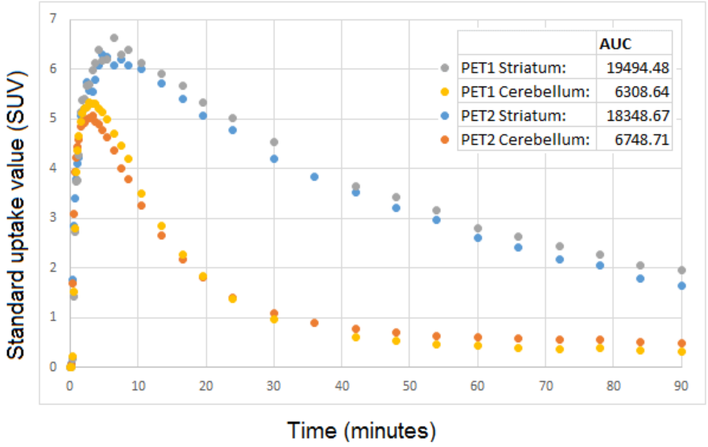

SUPPLEMENTARY FIGURE S3. SUV and AUC calculation for subject 8 to evaluate if an underestimation of the cerebellum due to mismatch of the attenuation map (of PET2; used for both PETs) might be the reason for higher BP_{ND} values in striatal areas of PET1 compared to PET2
